# Transforming Parkinson's Care in Africa (TraPCAf): protocol for a multimethodology National Institute for Health and Care Research Global Health Research Group project

**DOI:** 10.1186/s12883-023-03414-0

**Published:** 2023-10-19

**Authors:** R. Walker, N. Fothergill-Misbah, S. Kariuki, O. Ojo, R. Cilia, M. C. J. Dekker, O. Agabi, A. Akpalu, F. Amod, M. Breckons, M. Cham, S. Del Din, C. Dotchin, S. Guggsa, J. Kwasa, D. Mushi, F. O. Nwaokorie, T. Park, L. Rochester, J. Rogathi, F. S. Sarfo, A. Shalash, L. Ternent, S. Urasa, N. Okubadejo

**Affiliations:** 1 Northumbria Healthcare NHS Foundation Trust, Newcastle upon Tyne, UK; 2 Population Health Sciences Institute, Newcastle University, Newcastle upon Tyne, UK; 3 Neuroscience Unit, KEMRI-Wellcome Trust Research Programme, Kilifi, Kenya; 4 College of Medicine, University of Lagos, Lagos, Nigeria; 5 Lagos University Teaching Hospital, Lagos, Nigeria; 6 Parkinson and Movement Disorders Unit, Fondazione IRCCS Istituto Neurologico Carlo Besta, Milan, Italy; 7 Kilimanjaro Christian Medical Centre, Moshi, Tanzania; 8 Kilimanjaro Christian Medical University College, Moshi, Tanzania; 9 University of Ghana Medical School, Korle Bu Teaching Hospital, Accra, Ghana; 10 University of KwaZulu-Natal, Durban, South Africa; 11 Richard Novati Catholic Hospital, Sogakope, Ghana; 12 Translational and Clinical Research Institute, Faculty of Medical Sciences, Newcastle University, Newcastle upon Tyne, UK; 13 National Institute for Health and Care Research (NIHR) Newcastle Biomedical Research Centre (BRC), Newcastle University and Newcastle upon Tyne Hospitals NHS Foundation Trust, Newcastle upon Tyne, UK; 14 Addis Ababa University, Addis Ababa, Ethiopia; 15 Department of Clinical Medicine and Therapeutics, University of Nairobi, Nairobi, Kenya; 16 Institute of Public Health, Kilimanjaro Christian Medical University College, Moshi, Tanzania; 17 Department of Medical Laboratory Science, Faculty of Basic Medical Sciences, College of Medicine, University of Lagos, Lagos, Nigeria; 18 Parkinson’s Africa, Kingston upon Thames, UK; 19 Kwame Nkrumah University of Science & Technology, Kumasi, Ghana; 20 Department of Neurology, Faculty of Medicine, Ain Shams University, Cairo, Egypt

**Keywords:** Parkinson’s disease, Africa, Diagnosis, Treatment, Epidemiology, Microbiome, Metabolome, Pesticides, Genetics, Prevalence

## Abstract

**Background:**

Parkinson’s disease (PD) is the second most common neurodegenerative disorder and, according to the Global Burden of Disease estimates in 2015, was the fastest growing neurological disorder globally with respect to associated prevalence, disability, and deaths. Information regarding the awareness, diagnosis, phenotypic characteristics, epidemiology, prevalence, risk factors, treatment, economic impact and lived experiences of people with PD from the African perspective is relatively sparse in contrast to the developed world, and much remains to be learned from, and about, the continent.

**Methods:**

Transforming Parkinson’s Care in Africa (TraPCAf) is a multi-faceted, mixed-methods, multi-national research grant. The study design includes multiple sub-studies, combining observational (qualitative and quantitative) approaches for the epidemiological, clinical, risk factor and lived experience components, as appropriate, and interventional methods (clinical trial component). The aim of TraPCAf is to describe and gain a better understanding of the current situation of PD in Africa. The countries included in this National Institute for Health and Care Research (NIHR) Global Health Research Group (Egypt, Ethiopia, Ghana, Kenya, Nigeria, South Africa and Tanzania) represent diverse African geographies and genetic profiles, with differing resources, healthcare systems, health and social protection schemes, and policies. The research team is composed of experts in the field with vast experience in PD, jointly led by a UK-based and Africa-based investigator.

**Discussion:**

Despite the increasing prevalence of PD globally, robust data on the disease from Africa are lacking. Existing data point towards the poor awareness of PD and other neurological disorders on the continent and subsequent challenges with stigma, and limited access to affordable services and medication. This multi-site study will be the first of its kind in Africa. The data collected across the proposed sub-studies will provide novel and conclusive insights into the situation of PD. The selected country sites will allow for useful comparisons and make results relevant to other low- and middle-income countries. This grant is timely, as global recognition of PD and the public health challenge it poses builds. The work will contribute to broader initiatives, including the World Health Organization’s Intersectoral global action plan on epilepsy and other neurological disorders.

**Trial registration:**

https://doi.org/10.1186/ISRCTN77014546.

**Supplementary Information:**

The online version contains supplementary material available at 10.1186/s12883-023-03414-0.

## Background

The population of Africa is growing and ageing faster than any other region in the world [1]. The African population aged 65 + is predicted to grow to 570 million by 2100 (for context, the population of all high-income nations of the world is projected to reach 387 million by 2100) [1]. Sub-Saharan African countries will contribute to over half of the global population increase through 2050. This population expansion and demographic shift is inadvertently accompanied by an increase in the burden of non-communicable ageing-related neurological diseases such as Parkinson’s disease (PD) and dementia [2]. Yet ageing, ageing-related issues, and chronic conditions associated with later life, are not adequately prioritised by most African countries. This is despite the abundance of evidence indicating the significant impact (e.g., disability and dependency) attributable to such conditions, including neurodegenerative diseases [2, 3]. PD is the second most common neurodegenerative disorder and, according to the Global Burden of Disease estimates in 2015, was the fastest growing neurological disorder globally with respect to the associated prevalence [4], disability and deaths [5]. The high burden is fuelled by increasing longevity, the effects of industrialisation and environmental factors (for instance, pesticides, air pollution and solvents), and genetic susceptibility [6].

PD demonstrates significant heterogeneity in its causation (e.g., genetic, and environmental risk factors), access to specialised care and treatments and, inadvertently, quality of life and outcomes. Health disparities can affect patient outcomes negatively, and the social impact may vary across cultures [7, 8]. Information regarding the awareness, diagnosis, phenotypic characteristics, epidemiology, prevalence, risk factors, treatment, economic impact and lived experiences of people with PD from the African perspective is relatively sparse in contrast to the developed world, and much remains to be learned from, and about, the continent [7–12].

There have been few PD prevalence studies (most from several decades ago) and no community-based incidence studies in sub-Saharan Africa (SSA) [13–15]. Methodological flaws including the small population sizes surveyed and preponderant reliance on hospital-based data have given the erroneous impression that PD is rare in Africa. The recently published World Health Organization (WHO) technical brief, +‘Parkinson disease: a public health approach’, [16] alludes to the inconsistent data available on PD from low- and middle-income countries (LMICs), and the consequent difficulty of estimating the true burden in Africa or providing convincing data to motivate governmental policy makers and donor agencies to prioritise PD on the continent. Better understanding of the prevalence of PD in communities would justify prioritising PD, inform national policies, improve awareness of the disease, improve diagnostic rates, and access to health services, and drive the focus on providing access to pharmacological and non-pharmacological treatments (e.g., physiotherapy). Until now, there has only been one large scale prevalence study of PD in SSA, based on a house-to-house survey in Hai, Tanzania, in which less than a quarter of people with PD (PwP) had previously been diagnosed [17]. Furthermore, identifying the challenges and needs of PwP and their families in different countries and contexts [7, 8] would drive the narrative for local evidence-based policies [3].

Africa faces a stark gap in the provision of appropriate neurological workforces and trained physicians [18], care, rehabilitation and treatment, making life for PwP very difficult [19]. Awareness about PD is generally very low among the population [8, 20, 21] and healthcare professionals, resulting in under- and delayed diagnosis [10]. Exploring efficient and effective methods for diagnosing PD [22] (including use of telemedicine [23, 24] and/or novel biomarkers) could facilitate diagnosis at the primary care level, while offering opportunities for early management [16]. Sustainable medication is largely inaccessible and unaffordable across the continent [18, 25–28], however, investigations into alternative sources of levodopa (e.g., *Mucuna Pruriens* (MP)) have yielded positive results [29, 30].

Exploring pathophysiological mechanisms and the genetic aetiology of PD has the potential to improve understanding of susceptibility, environmental clustering and response to medication [9]. The developments in genome wide association studies (GWAS) have largely excluded genetically diverse populations [31]. A GWAS of PD among black Africans could provide a valuable source for finding novel genetic determinants that will inform new therapeutics that may eventually benefit patients from all populations [32, 33]. Mutations in genes commonly associated with PD, such as the Leucine Rich Repeat Kinase 2 (*LRRK2*) gene have demonstrated considerable regional variability, and have been virtually absent in black Africans from sub-Saharan Africa in studies reported to date [34–37]. This suggests the strong possibility that other yet undiscovered genetic factors may be at play. For example, the recently identified novel African-ancestry genetic risk factor in *GBA1* [38]. Identifying environmental risks for PD is also crucial [39, 40], and may identify a pathway to implement policy change to reduce exposure. PD is increasingly being referred to as a ‘man-made’ disease [41], with pesticides [42, 43] receiving substantial attention as an environmental risk factor for the development of PD. There is also strong evidence for important metabolic interactions between the gut microbiome, the gut, the immune system, and the brain [44–48].

Considering the evidence presented, this study protocol outlines the United Kingdom (UK) National Institute for Health and Care Research (NIHR) Global Health Research Group (GHRG) on Transforming Parkinson’s Care in Africa (TraPCAf) (Award No. NIHR133391). The NIHR is a major funder of high-quality global health research that addresses the diverse health needs of people in LMICs and supports the objectives of the UK Aid Strategy and the United Nation’s Sustainable Development Goals (SDGs) 2015–2030. The overarching aim of this research grant is to transform the landscape of PD diagnosis, treatment, and care across Africa. The protocol can also be accessed through the ISRCTN registry (https://doi.org/10.1186/ISRCTN77014546), where updates on the progress of the study will be documented.

## Methods

TraPCAf is a multi-faceted, mixed-methods, multi-national research grant. We report the study methods and design using an adaptation of the Standard Protocol Items: Recommendations for Interventional Trials (SPIRIT) guidelines [49, 50]. The study design includes multiple study aspects, combining observational (qualitative and quantitative) approaches for the epidemiological, clinical, risk factor and lived experience components, as appropriate, and interventional methods (clinical trial component). A cross-sectional cohort design is employed for the observational components, while a double-blind randomised controlled trial design is used for the interventional component.

### Aim and research questions

The aim of TraPCAf is to describe and gain a better understanding of the current situation of PD in Africa, addressing the following seven key research questions:

1. What is the prevalence of PD in Africa?
2. Are there unique risk factors (genetic and/or environmental) for PD in Africa and are there lessons about PD in general that we can learn from the African perspective?
3. How can rates of PD diagnosis be improved?
4. Does the clinical phenotype of PD in Africa differ from what has been reported in other populations?
5. How can the quality of PD management in Africa be improved?
6. How can affordable and sustainable treatment for PD be provided in Africa?
7. What is the current lived experience of people with PD, and their families, in Africa?

### Study setting

**Fig. 1:**
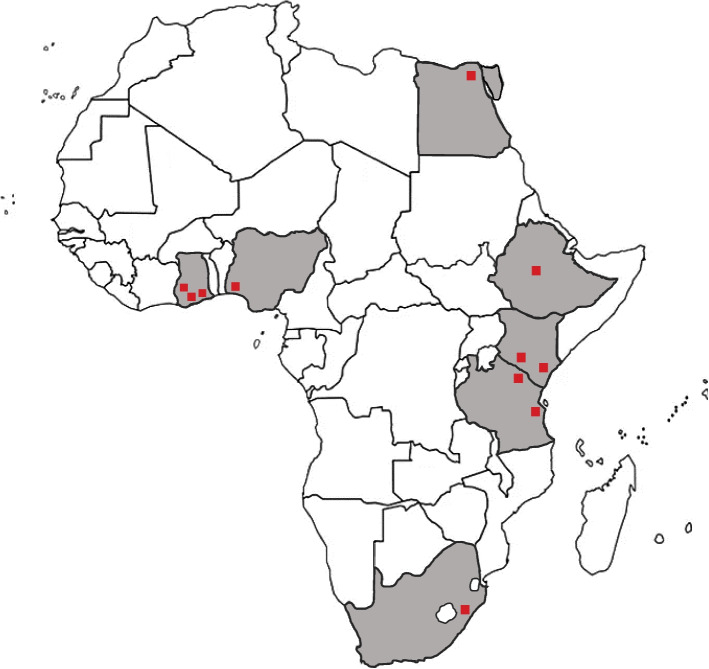
TraPCAf participating countries and sites marked with red box

Seven countries are included in this NIHR GHRG (Egypt, Ethiopia, Ghana, Kenya, Nigeria, South Africa and Tanzania) (Fig. 1) representing diverse African geographies and genetic profiles, with differing resources, healthcare systems, health and social protection schemes, and policies. This multi-country study will be carried out in two research settings, namely within the community and in hospitals (neurology clinics).

All seven countries will be involved in outpatient recruitment (see Table 1 for country sites). These research sites were selected based on the track record of research active clinicians, with an interest in PD, able to identify eligible individuals attending outpatient clinics. All hospitals included in the study are government, university-affiliated, or charitable organisations (not private), capturing data from most of the population.

**Table 1:** TraPCAf participating sites and outpatient locations

Country	Location	Outpatient site
Egypt	Cairo	Ain Shams University Hospital
Ethiopia	Addis Ababa	Tikur Anbessa Specialised Hospital
Ghana	Accra	Korle Bu Teaching Hospital
	Kumasi	Komfo Anokye Teaching Hospital
	Sogakope	Richard Novati Catholic Hospital
Kenya	Nairobi	Kenyatta National Hospital
Nigeria	Lagos	College of Medicine, University of Lagos
South Africa	Durban	Inkosi Albert Luthuli Central Hospital
Tanzania	Moshi	Kilimanjaro Christian Medical Centre
	Dar es Salaam	Muhimbili Mloganzila Hospital

The prevalence studies will be conducted in Tanzania, Kenya, Nigeria, and Ghana (as described below), selected based on the lack of epidemiological studies, the availability of infrastructure and delineated populations for a prevalence study (i.e., existence of a demographic surveillance system or a known denominator population in representative rural/urban populations) and the facilities and staff with prior experience to undertake a house-to-house survey.

### Study design

The study involves multiple study aspects (Table 2) that will be implemented across the seven countries (Table 1) in order to address the seven research questions above. In all seven countries, participants will be recruited through neurology outpatient clinics. In 4 countries (Ghana, Kenya, Nigeria, and Tanzania) participants will also be recruited through community-based prevalence studies.

**Table 2:** Overview of TraPCAf sub-studies

Research question	Outline of work	Study component	Study details
1. What is the prevalence of PD in Africa?	Community, door-to-door prevalence studies in 4 sites	Door-to-door household survey and clinical exam	Community screening and clinical examination.
2. Are there unique risk factors (genetic and/or environmental) for PD in Africa and are there lessons about PD in general that we can learn from the African perspective?	Exploring the biological mechanisms of PD, conducting genome-wide association and exploring the role of the microbiome and pesticide exposure in PD	a) DNA analysis	Collection and analysis of blood and saliva samples.
		b) Microbiome and metabolite analysis	Collection and analysis of stool and buccal (cheek) samples.
		c) Pesticide, heavy metal and trichloroethylene analysis	Collection and analysis of water, soil, and urine samples.
3. How can rates of PD diagnosis be improved?	Development and assessment of aids to diagnosis, including use of screening tools, technologies for gait assessments, and the role of biological samples as possible diagnostic tools	a) Biological diagnostic tools	Collection and analysis of alpha synuclein in blood plasma, blood serum and saliva samples. Collection and analysis of sebum samples.
		b) Technology diagnostic tools	Collection and analysis of gait assessments. Collection and analysis of neuromotor pen data. Collection and analysis of Optical Computerised Tomography (OCT) data. Collection and analysis of smartphone application data.
4. Does the clinical phenotype of PD in Africa differ from what has been reported in other populations?	Phenotype PD using clinically validated scales and understand progression of PD by following up PwP in outpatient settings	a) Clinical assessment	Clinical examination.
		b) Technology for monitoring progression	Collection and analysis of gait assessments. Collection and analysis of smartphone application data.
5. How can the quality of PD management in Africa be improved?	Understanding current management and improving the management of PD through training	a) Economic evaluation	Economic evaluation of current care and services.
		b) Capacity building	Understanding the role of video examination and telemedicine in PD care. Training of healthcare professionals.
6. How can affordable and sustainable treatment for PD be provided in Africa?	Comparing *Mucuna Pruriens* versus levodopa/carbidopa for the treatment of PD, including an economic evaluation to assess cost-effectiveness	Randomised controlled trial (RCT)	Randomisation to MP or levodopa/carbidopa. Economic evaluation.
7. What is the current lived experience of people with PD in Africa?	Exploring the lived experience of PD through qualitative interviews, the development of information packages and educational campaigns, and the establishment of support groups	a) Qualitative interviews	Analysis of interviews with PwP, family members and stakeholders.
		b) Community education	Development and evaluation of information packages. Development and evaluation of educational campaigns.
		c) Support groups	Development and evaluation of support groups for PD.

#### Processes for recruitment

PwP identified through outpatient clinics, and through prevalence studies, will be invited to participate in TraPCAf, will be provided with information sheets on each of the different aspects of the research (see Table 2), and be able to consent to particular study aspects. The clinical assessment (part of RQ4) is the minimum involvement a participant can have. Consenting participants can, but do not need to, participate in all study aspects. Furthermore, 2 healthy population controls of the same ethnicity, matched for gender and age will be recruited for every 1 PwP, providing the same biological and environmental samples as PwP (RQ2 and RQ3), and undergoing the same clinical assessments (RQ4).

The inclusion criteria for the prevalence studies are:

- 1. Consenting resident in the delineated community for at least 12 months prior to the date of survey.
- 2. Age 18 years or older for Tanzania, Kenya, Ghana (no upper limit) and > 40 for urban Lagos site.

Inclusion criteria for PwP in all studies are:

- 1. Consenting adults aged 18 years or older (no upper limit) diagnosed with PD based on UK Parkinson’s Disease Brain Bank (UKPDBB) clinical diagnostic criteria [51] of any stage.
- 2. For the *Mucuna Pruriens* trial, PwP should be treatment naïve (summary of inclusion criteria detailed in RQ6 section below).

Inclusion criteria for healthy controls are:

- 1. Consenting healthy, neurologically normal (assessed at in-person physical examination) adults aged 18 years or older (no upper limit) who are age, gender and ethnicity matched (per country).

Prospective participants will be excluded if they do not consent or lack capacity to consent (clinical judgment), and/or are physically unable to complete study procedures due to advanced disease and physical disability. Participants will provide details of medical comorbidities, if known, during the clinical examination.

### Group establishment

Group establishment began before the awarding of the grant, during the proposal development phase. This involved bringing together experts from across the seven partner countries to develop the aims, research questions and study methods of the grant. Importantly, this development was guided by our community engagement and involvement (CEI) team.

CEI work ensured the research proposal was designed with, and for, people with PwP and their families. A 2 hour virtual CEI meeting was held with 16 representatives from Nigeria, Kenya, Ghana, and Ethiopia during proposal development to understand their needs, challenges and priorities, their interest and willingness to participate, introduce the proposed research and solicit feedback on the relevance, approach, applicability and objectives. For example, the research team highlighted the challenge of diagnosing people in a prevalence study in contexts where there may not be access to affordable or sustainable drug treatment. However, PwP felt that knowing a diagnosis would give people a better chance to manage the disease, avoiding perceptions associated with witchcraft. Considering this, we are confident that understanding the prevalence of PD across the sites is vital, and knowing a diagnosis may outweigh the challenges of medication accessibility. The CEI team also echoed the need for information about PD burden to push for local policy changes.

Post-award, the group establishment phase has involved ethics application submissions, mapping research priorities, acquiring necessary consumables, a review of existing literature on PD from participating countries, and the planning of CEI activities in-country. The role of CEI in this project is to improve the equality, efficacy and impact of responses and services by ensuring that PwP are active stakeholders in the deliberations, design, decision-making, implementation, and dissemination of the project. This will be monitored using the Community Engagement Process Index. Working with CEI representatives from each country ensures that our engagement is context specific and localised, taking into account national and regional considerations, while recognising similarities and trends across communities and nations.

### Study components

The following sections describe the details of the studies outlined in Table 2*,* addressing research questions 1–7.

All biological samples will be collected by a research nurse at a clinic visit, unless otherwise indicated. All sites have a material transfer agreement with Newcastle University and collected samples will be shipped to Newcastle University for analysis in labs with relevant expertise. Sites have the option to aliquot samples for analysis in situ and for capacity building with expert support and training.

#### RQ1. Prevalence studies in Ghana, Kenya, Nigeria, and Tanzania

To estimate prevalence of PD and/or parkinsonism in Ghana (South Tongu district, Volta region, population 113,114), Kenya (Kilifi, population 300,000), Nigeria (Lagos, subset of population of 1.8 million), and Tanzania (Hai region, population 180,000), door-to-door household screening will be carried out by local, trained health workers in their designated areas/villages. Information recorded will include village name and precise geographical location, head of household, date of visit, name of household member, age, and sex. The head of household will report on behalf of the household (if they are not present, anyone over the age of 18 will be able to respond on the household’s behalf). Data on PD will be collected using an adapted screening tool (from methodology initially described by Dotchin et al. (2008) in Tanzania [17]) as follows:

1. Do you walk more slowly than other people your age?
2. Do your arms or legs shake?
3. Do you shuffle or take short steps when you walk?
4. Do you fall easily?
5. Has anyone told you that you have Parkinson’s disease?

Any positive responders will be reviewed by a study clinician in the community to determine likely diagnosis and advised appropriately. If it is thought they might have PD, they will be invited to an in-person physician assessment and neurology examination to determine diagnosis. Those found not to have PD (or other neurological condition) will be provided with referral advice to an appropriate healthcare facility. Those diagnosed with a neurological condition will be registered at the local clinic for follow-up care. All individuals diagnosed with PD during the prevalence studies will be invited to participate in further study aspects.

The primary outcome measures for the prevalence study will be the number of people with idiopathic PD per 100,000 of the population studied in each country [52], with age standardisation to the WHO population. We will also be reporting on the number of vascular parkinsonism cases per 100,000 population – in view of the high rates of undiagnosed and untreated hypertension in SSA, we expect there may be high rates of vascular parkinsonism. The secondary outcome measure will be sex-specific prevalence of PD in Africa (measured as number of male and female per 100,000 population), again with age-standardisation.

#### RQ2. Risk factors for PD

- **a) DNA analysis**
  TraPCAf recruitment will link with the Global Parkinson’s Genetic Program (GP2) (www.gp2.org) and the Aligning Science Across Parkinson’s (ASAP) initiative (www.parkinsonsroadmap.org). The ASAP initiative is devoted to accelerating the pace of discovery and informing the path to a cure for PD through collaboration, research-enabling resources, and data sharing. GP2 is a resource programme of the ASAP initiative focused on improving understanding of the genetic architecture of PD and making this knowledge globally relevant.
  In summary, genome-wide association studies and related genomics explorations will be conducted utilising DNA extracted from whole blood and/or saliva samples. The aim of this collaboration is to contribute 1000 samples across TraPCAf sites (plus 2000 samples from healthy controls), which will be shared with GP2 for analysis and sequencing, contributing to GP2’s goal to genotype more than 150,000 unique samples from diverse populations.
  We will work closely with our CEI team to determine how best to return genetics results for variants whose significance is known (i.e., clinically relevant information) to PwP and understand what follow up and counselling is available in-country. Where expertise is lacking, we will work with GP2 to build capacity in genetic counselling. Anonymised genotypic data will be linked with phenotypic data and microbiome analyses. The primary outcome will be the Odds ratio (or Hazards ratio) of specified single nucleotide polymorphisms (SNPs) measured as the frequency of polymorphic variation in PwP versus controls (based on the genotyping results from the NeuroChip® [53] genotyping platform). Genome-wide analysis data comparing PD and controls will also be reported as an outcome by the TraPCAf research team in collaboration with GP2.
- **b) Microbiome and metabolite analysis**
  Stool samples and buccal swab samples will be collected using standardised equipment across all sites from all consenting participants for 16S sequencing for microbiome analysis and metabolite analysis [54]. All datasets obtained will be identified to bacterial species-level operational taxonomic units (OTUs) to differentiate the faecal community composition of individuals from the rural and urban communities under study. Analysis pipeline to capture microbiota data on alpha diversity, beta diversity, differential abundance of microbial taxa and functional gene will be utilised [54]. Primary outcome (conducted at baseline) will be the difference between cases and controls. In conjunction with microbiome and metabolite analysis, a questionnaire on diet and medication use (see supplementary information) developed for the purposes of addressing TraPCAf research questions will be administered. Samples will be linked to individuals’ genotypic and phenotypic data to allow for further analysis.
- **c) Pesticide, heavy metal and trichloroethylene analysis**
  To estimate the impact of environmental exposures on the development of PD, water and soil samples will be collected using standardised procedures from the homes of identified participants who consent. This will primarily involve participants recruited through the prevalence study but those attending outpatient clinics will also be invited to participate in this study aspect and given the equipment and instructions to collect and bring samples from their homes to their next clinic visit. Samples will be analysed for pesticide content, heavy metal content and the presence of trichloroethylene. In conjunction with the analysis, the mini-environmental risk factor questionnaire (MERQ-PD) [55] will be administered in a case–control design and analysed alongside pesticide data from collected samples. A pesticide and heavy metal exposure questionnaire, developed by the TraPCAf team for the purposes of addressing the research question (supplementary material), will also be administered to understand historical toxin exposure.
  Urine samples will also be collected from consenting participants at the clinic visit and analysed for pesticide residue levels to determine individual exposure. Levels will be compared to previously published results indicating potentially dangerous exposure. Inclusion of urine samples will provide an indication of body burden but also of recent and continued exposure levels. Environmental risk factors for PD will be measured at baseline using (i) toxicology assessment for contaminants of emerging concern related to PD (including pesticides, herbicides, other chemicals) in the soil and water samples at residences of PwP versus healthy controls and (ii) comparison of the risk exposures of PwP and controls based on responses in the MERQ-PD questionnaires and reported as the relative risk of exposure for each subcategory of risk factors interrogated.

#### RQ3. Aids to diagnosis for PD

We will explore the diagnostic utility (predictive values) of several innovative methods for improving the clinical diagnosis of PD. These include the use of biological markers, as well as technological methods (see Table 2). Primary outcome measures for the sub-studies exploring aids to diagnosis will include the diagnostic utility, positive predictive value, accuracy of diagnosis of PD versus normal control, area under Receiver Operating Characteristics (ROC) curve, negative predictive values, sensitivity, and specificity of the diagnostic modalities described. For all study aspects within this RQ, consenting participants will be invited to provide samples, or to trial the technology tools.

- **a) Biological diagnostic tools**
  **Plasma, serum, saliva, and urine analysis** Aggregated alpha-synuclein in brain and peripheral nervous system tissue is a diagnostic feature of PD. We are collaborating with a team at Newcastle University, through registered partner company ‘ESP Diagnostics’ (www.espdiagnostics.com) who have developed an assay for extra-cellular synucleinopathic protein (ESP), that detects recombinant alpha-synuclein aggregation using a fluorescent dye (covered by patent rights (WO2019/171035A10)). This works on urine, blood plasma and blood serum samples, requiring equipment available in most hospital biochemistry laboratories (for further information on the sensitivity and specificity using saliva see [56, 57]). Blood will be taken using standard blood-taking equipment by a research nurse. For saliva, the participant will spit into a tube. Urine will be collected in a plastic beaker. All samples will then be prepared, aliquoted and stored appropriately.
  **Metabolome analysis** Metabolomics is the analysis of small molecule metabolites contained within cell, tissue and biofluid systems. PwP may have a particular odour, and this is currently being investigated at the University of Manchester (www.mbc.manchester.ac.uk/barrangroup). We are collaborating with the team leading this work to investigate whether metabolites can be used to diagnose PD [22, 58] (covered by patent rights US20230077659A1 and US20230080918A1). The team have developed a diagnostic platform, using mass spectrometry, that can classify PD from sebum samples with up to 78–88% accuracy (for detailed methodology see [22, 59]). Briefly, sebum samples will be taken by a research nurse with a cotton bud rubbed on the upper back (a sebum-rich area of the body) for metabolite analysis. Primary outcomes for this analysis (conducted at baseline) will be the diagnostic utility of skin metabolites measured in sebum in differentiating PwP from controls. Metabolome samples will also be linked with phenotypic and genotypic data. It is important to determine whether the “PD signature” identified is a pure disease-related signature and will be detected in Africans with PD, despite genetic, epidemiological, and dietary differences. Findings will confirm the more widespread utility of swabs as a methodology to help diagnose PD and provide a unique window into underlying pathophysiology. This could be particularly useful in Africa, as samples remain stable at room temperature for several weeks, facilitating storage and transport.
- **b) Technology diagnostic tools**
  **Digital mobility outcomes** Gait assessment can discriminate and predict early disease, track disease progression, and identify therapeutic response in PD [60]. Wearable technology (such as accelerometers and inertial measurements units (IMUs)) offer a low-cost, unobtrusive solution to record the signals needed to extract comprehensive gait features in laboratory and real-world scenarios. Widespread development and adoption of commercial sensors by the public for activity monitoring demonstrates their feasibility and acceptability in HICs, but these technologies have been under-utilised in LMICs to date. Axivity AX6, a low-cost non-invasive wearable device (www.axivity.com/product/ax6) which is a data logger capable of recording raw data from a suite of integrated sensors) [61, 62] will be used to objectively monitor mobility (walking activity and gait) during free-living mobility in PwP. This study aspect will initially be rolled out to participants in Ghana, and potentially other sites based on initial outcomes. Briefly, the sensor is secured to the participant’s lower back (see [61] for image of placement) who will be advised to continue with their usual activities and not change their routine, with monitoring over 7 days [63]. The device quantifies digital mobility based on walking speed, step count, walking bout number and duration, for example. The device has previously been validated in the UK [62, 64] and piloted in people with frailty in Tanzania [61], demonstrating feasibility. Based on previous estimates (pilot in Tanzania), we will initially recruit 50 PwP from the Ghana sites. Gait assessments will also be used to monitor disease progression as part of RQ4.
  **NeuroMotor Pen (NMP)** The NMP is a hand-held medical device, developed by ‘Manus Neurodynamica’ (www.manusneuro.com), with a unique patented system combining sensor technologies in a digital pen with software and an analytical engine with Decision Support System. The interface enables users to non-invasively record parameters of minute limb and hand motion from graphical tasks and apply automated analysis, with high reproducibility [65]. These parameters are used as ‘digital biomarkers’ to provide objective information about movement abnormalities. The NMP, designed with a specific intended use in the clinic, assesses and quantifies motor skills based on simple copying (writing and drawing) tasks that are not dependent on literacy. Unlike wearables, the NMP measures subtle symptoms that cannot easily be seen or quantified with the naked eye and provides a test of the extrapyramidal system. Symptoms currently recorded with the UPDRS, such as bradykinesia and tremor, can be objectively quantified with NMP. Previous research suggests that the NMP is a quick, inexpensive, non-invasive and objective aid to diagnosis [66]. The NMP has undergone two phases of clinical validation in European cohorts with high differential diagnostic sensitivity (PD vs not PD) (80%) and high specificity (75%). This technology will be initially piloted with participants in 2 sites, and rolled out to additional sites after the feasibility of use in lower resource settings is better understood.
  **Optical coherence tomography (OCT) imaging** People with confirmed and prodromal PD have been reported to have changes in retinal structure [67]. Retinal imaging via OCT is a quick, non-contact, inexpensive method to detect these changes. It is an infra-red based imaging technology with no eye drops required, making it well tolerated. Yet, retinal changes have yet to be used to detect neurological disease or monitor progression [68]. In this work, we propose to build on advanced statistical models developed by our collaborators in the ‘OCTAHEDRON’ project at Newcastle University (www.research.ncl.ac.uk/octahedron) to automatically identify biomarkers of PD diagnosis in retinal OCT scans [69]. Such tools have been developed using data mainly from the eyes of individuals in white populations from high-income countries. We aim to validate and tune these models to ensure they also serve the health needs of LMIC populations [70]. The increased accessibility of OCT imaging in lower resource settings highlights the relevance for diagnostic and disease monitoring. In addition, data collected will allow the exploration of biomarkers for present and future disease progression to maximise the impact of limited clinical resources. All sites with this equipment available, and with the necessary expertise to carry out retinal imaging, will be able to enrol participants to this study aspect.
  **Oxford smartphone application** The ‘Oxford Parkinson’s Disease Centre’ in the UK (www.dpag.ox.ac.uk/opdc) have piloted, tested and refined a 6-min smartphone test of motor symptoms in 522 participants with early PD, prodromal Rapid Eye Movement (REM) sleep behaviour disorder (RBD) and age-matched controls, showing accuracies of 85–92% in distinguishing PD versus control and RBD versus PD [71]. Using novel machine learning algorithm MLA data approaches, they can use this smartphone test to predict motor Unified Parkinson’s Disease Rating Scale (UPDRS) with a high level of accuracy, and to predict clinically relevant change, such as the onset of falls, 18 months before occurrence, with positive predictive values around 90% [72]. The applicability of this smartphone application to the African context will be initially tested in 4 sites, and rolled out to additional sites once usability is better understood. The app could be used to accurately distinguish PD from controls (and PD from idiopathic REM sleep behaviour disorder), providing a growing consensus for the utility of digital biomarkers in early and prodromal PD, which could be of particular use in settings where specialised clinicians are lacking. The smartphone application will also be used to monitor progression as part of RQ4

#### RQ4. Clinical phenotype and progression of PD

- **a) Clinical assessment**
  All PwP, either previously or newly diagnosed from each site, will be invited to a detailed examination and assessment using standard disease specific questionnaires, e.g., the International Parkinson and Movement Disorders Society Unified PD Rating Scale (MDS-UPDRS) (see Table 3), in addition to study-specific questionnaires developed by the research team (see supplementary material). At yearly intervals we will follow up these cohorts, including monitoring for disease progression (MDS UPDRS and Hoehn and Yahr scale) and cognitive decline (using Intervention for Dementia in Elderly Africans (IDEA) and Montreal Cognitive Assessment (MoCA) scores), assessing response to treatment, reviewing compliance with medication, and assessing motor symptoms (MDS-UPDRS total and Part III scores) and non-motor symptoms (MDS-NMS) [73].

**Table 3:** Data collection tools to be administered during clinical examination and assessment

Tools and questionnaires
Clinical observations^a^
Neurology assessment^a^
Questionnaire on women’s symptoms^a,b^
Healthcare resource use questionnaire^a,c^
MDS Unified PD Rating Scale (**MDS-UPDRS**) [74]
Clinical impression of severity index (**CISI-PD**) [75]
MDS Non-Motor Symptoms Scale (**NMSS**) [76]
Barthel index [77]
Intervention for Dementia in Elderly Africans (**IDEA**) cognitive screening tool [78]
Mini-environmental risk factor questionnaire (**MERQ-PD**) [55]
Questionnaire on pesticide exposure^a^
Questionnaire on microbiome^a^
PD Questionnaire (**PDQ-8**) [79]
EuroQol- 5 Dimension instrument (**EQ5D-5L**) [80]
REM sleep behaviour disorder questionnaire (**RBDSQ**) [81]
Hospital Anxiety and Depression Scale (**HADS**) [82]
Montreal Cognitive Assessment (**MoCA**) [83]
PD Sleep Scale (**PDSS**) [84]
Epworth Sleepiness Scale (**ESS**) [85]
Questionnaire for impulsive-compulsive disorders in PD – Rating Scale (**QUIP-RS**) [86]
Scales for outcomes in PD – Autonomic (**SCOPA-AUT**) [87]

^a^Unvalidated questionnaires developed (based on expertise) for the purposes of addressing TraPCAf research questions (see supplementary material)

^b^Questionnaire developed and adapted from the Fox insight ‘Experience of women with PD’ survey [88]

^c^Questionnaire developed and adapted from the ‘Resource Utilization in Dementia’ (RUD) instrument [89]
  A PD registry of participants at each site will be established, with consent to contact and contact information (including phone numbers and next of kin contact). Participants will be able to consent to follow-up in the future, i.e., beyond the duration of the grant. In line with this, we will be asking participants to consent to being followed up and have their contact details kept for 20 years after the study end date, with the potential to follow up the cohort.
- **b) Technology for monitoring progression**
  The digital mobility outcomes and Oxford smartphone application described in ‘Technology diagnostic tools’ (RQ3) will also be used to monitor disease progression.

#### RQ5. Improving PD management in Africa

- **a) Economic evaluation**
  An economic evaluation, involving a mapping exercise of current care provision for PD as well as modelling of alternatives to current care, will be carried out. This work will take the form of a cost consequence analysis of the diagnosis and management of PD, including use of formal health services, alternative healing (e.g., visits to traditional healers), patient related costs, days lost to care and the impact on economic productivity. These data will be collected as part of the modified RUD questionnaire (Table 3).
- **b) Capacity building**
  **Video examination and telemedicine** Telemedicine programmes are particularly suited to PD, and other movement disorders, primarily because much of the physical exam findings are visual [24]. Telemedicine can provide an accessible, cost-effective, and high-quality healthcare services [90], and presents as a promising avenue for the effective mobilisation and utilisation of the few neurologists in Africa. Within TraPCAf, video consults will be used to aid (confirm) diagnosis and determine feasibility in all sites. This draws on technology developed and trialled initially between Toronto and Lagos, and subsequently elsewhere, including Tanzania and Ghana as part of the African Section of the MDS. Scripted videos are uploaded securely to the internet for reporting by movement disorder specialists (see supplementary material). Video consults with scripted videos will be trialled across all sites and follow guidance detailed by Duker (2013) [91].
  **Training of healthcare professionals** Research and diagnostic capacity building have the potential to empower African neuroscientists and allied health professionals (AHPs) with the means to develop innovative strategies that address local needs, transfer relevant technology, and provide solutions to decrease burden and improve the quality of outcomes for PwP in Africa. Training and capacity building will increase the critical mass of African researchers with the ability to define research priorities, conduct epidemiological research, conduct a diversity of economic impact evaluations, service delivery appraisals, and strategies for prevention. Clinical trials and development of non-pharmacological evidence-based treatments remain a high priority. It is thus imperative to build a considerable pool of researchers and clinicians/healthcare providers proficient in conducting research and providing diagnostic and therapeutic services and with enough competency to participate meaningfully in collaborative international research, who will be the future leaders that will sustain PD research, education and clinical competency in Africa.
  A survey of the 7 participating countries will be conducted to identify training priorities among healthcare professionals. Capacity building initiatives will aim to train neurologists, geriatricians, nurses and AHPs in PD management as well as epidemiology, biostatistics, clinical trial methods, bioethics and grantsmanship. Ultimately, this will build multidisciplinary models of care for PD, informing policy and practice which could be replicated for the management of other chronic and age-related conditions, while also building capacity for programme development at a postgraduate level to ensure long-term training and research is enabled. The team will build on past experience running such educational events in Africa (in-person and virtually). For example, the International Parkinson and Movement Disorders Society (MDS) ‘Overview of Movement Disorders for Physicians, Nurses and Allied Health Professionals course’ held in Moshi, Tanzania in 2019, and the ‘MDS-Africa Nurse and Allied Health Professionals Course for Parkinson’s Disease’ online course held in 2021. The team will also utilise the expertise of collaborating organisations, for example, GP2.

#### RQ6. Randomised clinical trial of mucuna pruriens

*Mucuna Pruriens* (MP) is a levodopa-containing leguminous plant that grows wild in tropical regions of the world. Therefore, MP-based therapy has the potential to replace or supplement levodopa-based medicines in countries where levodopa is unaffordable and inaccessible due to the low costs of preparation of MP and high natural availability (up to 9.5% levodopa content) [29].

We will undertake a phase II, 12-month, 2-centre double-blind (participant and investigators), parallel-group, randomised controlled trial (RCT), only in Tanzania, addressing the drug naïveiority of MP versus levodopa/carbidopa (which is the reference treatment) for the treatment of PD in drug-naïve PwP. Medication will be prepared to comply with the double-blind design, i.e., mock tablets and mock MP flavoured powder. The trial will use methodology for the current multi-centre trial of MP in Ghana (trial registered on Cochrane Central Register of Controlled Trials (www.trialsearch.who.int/Trial2.aspx?TrialID=PACTR201611001882367)), where detailed methods can be found. Methods for the trial are briefly outlined here:

##### Inclusion criteria

(i) age range 30 to 80 years.
(ii) diagnosis of clinically probable or clinically established PD according to currently established criteria [92].
(iii) newly diagnosed PD < 2 years.
(iv) never treated with levodopa (drug-naïve) or treated for only ≤ 6 months during the disease course but Levodopa discontinued since at least 3 months.

##### Exclusion criteria

(i) dementia according to DSM-V criteria precluding the subject to provide written informed consent.
(ii) clinically significant psychiatric illness (e.g., severe depression or psychosis).
(iii) Hoehn and Yahr stage 5/5 [93].
(iv) severe, unstable medical conditions (e.g., neoplasms; unstable diabetes mellitus; heart, renal or liver failure).
(v) pregnancy.

##### Sample size

The sample size [52] sufficient to have a power of 80% with a two-tailed type-I error of 20% to detect clinically meaningful difference (effect size of 0.5 according to Cohen [94]) is 74 patients (37 per each intervention). A drop-out rate of 20% was considered for the final sample size calculation [95], resulting in 45 patients per treatment arm. Randomised allocation will be performed according to a computer-generated randomization list concealed by sealed envelopes.

##### Outcome measures

The primary outcome is the non-inferiority of MP as measured by motor UPDRS subsection score change over the time frame of the trial. The main secondary outcome is the summary index of the PDQ-8. Six assessments will take place at baseline, 1–3-6–9-12 months and at end of the study. The titration phase is 4 weeks in which levodopa dose will be slowly titrated up to 4.5 mg/kg/day in three doses. Patients are randomised either to 40 weeks treatment with MP powder or to 40 weeks treatment with levodopa/carbidopa tablets. An additional secondary outcome will be objective measuring motor performance using the Oxford smartphone app (detailed in 2.5.3).

##### Data handling

With regards to data handling, and to ensure privacy and confidentiality, all participants will be pseudonymised. To minimise bias, only the lead investigator will be authorised to input patient data. Data is password protected, for intended purposes only and will be kept confidential and stored securely in a lockable cabinet that can be assessed by the lead investigator.

##### Trial economic evaluation

An economic evaluation will be conducted alongside the MP trial, comprised of a cost-utility analysis based on the incremental cost per quality-adjusted life year (QALY) gained, based on the participants’ responses to the EQ5D-5L questionnaire sand PDQ8. Intervention and follow up costs will also be calculated from the perspective of formal health service used and additional costs incurred by patients. We aim to estimate the cost of the interventions, subsequent use of services and additional costs to participants and their families to inform the cost-effectiveness and cost–benefit of MP versus levodopa/carbidopa.

#### RQ7. Lived experience of PD

- **a) Qualitative study**
  A qualitative study to understand lived experiences of PD will be conducted across all 7 African sites. Semi-structured, audio-recorded interviews exploring diagnostic journeys, use of healthcare, care structures, experiences of stigma, among other emerging aspects, will be carried out with PwP and primary caregivers. A purposive sample of 20 PwP and 20 caregivers (with consent from PwP), totalling 360 participants, will be recruited from each site in order to get a representative sample of, for example, age, gender, disease stage, disease duration, socioeconomic status. Furthermore, healthcare professionals and policy makers will be invited to interview about their experience of PD (*n* = 20 per country). The primary outcome will be descriptions of lived experiences of PwP across, and within, different sites. We aim to understand what similarities populations experience with regards to PD, and where differences exist to inform future interventions to support PwP and caregivers in the community and inform policy to support their care.
- **b) Community education**
  Working closely with our CEI team, we will look at ways to increase awareness of PD in communities, utilising local media outlets, training events, and awareness-raising activities. The CEI team will lead on the development of culturally-specific information packages for PwP and caregivers in local languages (where resources are not available already). Ministries of Health will be involved from the outset to maximise the potential for translating research findings into practice. A needs assessment will guide the specific requirements of each site.
- **c) Support groups**
  Support groups play an important role in care and support for PwP and caregivers, while also filling in gaps in information and services that many health systems in low resource settings are unable to provide [96]. Therefore, PD support groups are important components within these settings, ensuring PwP and their families have the support they need, and a platform to advocate for PD. Support groups already exist in five of the seven countries we are working in. Support groups will be established in the two sites that do not already have groups (Egypt and Tanzania). Additional groups, focussed on more rural areas of our seven countries, will also be established. A virtual support group, facilitated by our CEI partners, Parkinson’s Africa, will also be made available to all PwP and caregivers involved in TraPCAf.

### Data analysis and management

A Research Electronic Data Capture (REDCap) database, hosted at Newcastle University (UK), has been built and will be used to store all questionnaire data across sites. Data will be analysed using standard statistical software packages (Statistical Package for Social Sciences (SPSS), company of manufacture). Biological and environmental samples will be linked to questionnaire data on REDCap through associated barcodes (biobank) and samples stored at Newcastle University for analysis, or onward transfer, as described. Technology data will be transferred and uploaded to secure cloud servers and processed by the technology owners (our collaborators).

Data will be summarised using descriptive statistics appropriate to the nature of the data (e.g., mean, median, standard deviation, inter-quartile range, frequency). Bivariate inferential tests and multivariable modelling will be used where appropriate. For the prevalence study, data will be presented as proportions of the denominator population, with sub-group analysis by sex and age bands. Confidence interval generation will assume a bivariate distribution. For the RCT, independent sample t-test will be applied, with either multilevel logistic regression or a mixed linear model during secondary analysis to adjust for baseline score, treatment site, demographic and disease factors not adjusted for by randomisation. For screening tools, accuracy will be compared to the gold standard of expert physician opinion. The primary measure of criterion validity will be area under the receiver operating characteristic (AUROC) curve. Sensitivity, specificity, and predictive values will also be reported. Qualitative data will be managed using NVivo qualitative data analysis computer software and analysis will be based on principles of Thematic Analysis [97]. Data will initially be coded using a mixture of deductive and inductive coding after which coded data will be analysed to identify key themes, which will emerge from the data (not pre-identified). Throughout the lifecycle of the project, CEI advisory groups will feed back to the research team on data collection tools, recruitment and be involved in preliminary analysis, as well as the development of appropriate methods for dissemination of findings to communities.

### Ethics approvals and consent to participate

Ethical approval for this study was granted by Faculty of Medical Sciences Research Ethics Committee, part of Newcastle University's Research Ethics Committee on 1st February 2023 (Application No. 2453/26903/2021). Ethical approval is already in place in Tanzania, Ghana and Nigeria at time of protocol publication, and will be in place in other sites before recruitment commences.

As stated in our ethical approval, all research participants will be required to give fully informed, written consent to participate in any study aspect. All potential participants will be given an information sheet and will be able to read the information (translated in local languages as appropriate) or have it read to them. Participants will have the opportunity to ask questions about the project and can consent to the aspects of the study they want to be involved with. Participants will not be taking part in any study aspect without knowing the details of the study and their participation rights. Those who are unable to write (e.g., illiterate) will be able to provide a thumb print on the consent form or have a witness append the form on their behalf, with verbal consent. Participants will also be able to consent to follow up in the clinical aspect of the study. The only situation where participants may not be able to give full informed consent is if they lack capacity; this will be for a very small group of individuals and is necessary for the prevalence studies. In these cases, consent will be obtained from their next of kin and we will gain their verbal assent. Their further involvement in the study will be minimal.

Participants will receive a debriefing sheet after their participation in the study, which will provide a lay summary of how the data collected will be stored and used to transform the Parkinson’s landscape in Africa, as well as reiterating their participation rights (e.g., right to withdraw until data have been aggregated for analysis and reporting) and contact details for the study team. If they consent, participants will also be linked with support groups in-country and our charity partner network.

## Discussion

Despite the increasing prevalence of PD globally, robust data on the disease from Africa are lacking. Existing data point towards the poor awareness of PD and other neurological disorders on the continent and subsequent challenges with stigma, and limited access to affordable services and medication.

This multi-site study will be the first of its kind in Africa. The research team is composed of experts in the field with vast experience in PD (both clinical and research), jointly led by a UK-based and Africa-based investigator. We anticipate that the data collected across the proposed studies will provide novel and conclusive insights into the situation of PD on the continent through a deep understanding of those already accessing care and services, and persons with PD in the community who may have never obtained a diagnosis. The selected country sites will allow for useful comparisons and make results relevant to other LMICs. The grant will also provide opportunities for further studies driven by researchers in the TraPCAf sites and beyond and build a platform for launching other collaborations including genomics and gene-environment studies and exploring the impact of CEI activities and longitudinal studies on outcomes (including patient-reported outcomes).

The limitations of the overall study mainly relate to the scope of the work to be conducted within allocated budgets. Efficient and effective communication across sites and within the research team is crucial to ensure positive outcomes. Furthermore, feedback and input from the CEI team, as well as our External Advisory Group, and funders, will ensure the grant’s success. We are aware of the issues relating to access to medicines, particularly for those who are diagnosed during the prevalence studies. Hence, the role of advocacy in tandem with the research is crucial to ensure that we build capacity and awareness in each country. We are particularly keen to enable access to affordable and sustainable medication and are working with WHO and charitable foundations in respect to this.

Although the seven African countries broadly represent different regions of the continent, we are necessarily limited to forging relationships and networks with active neurologists and researchers. Findings from this grant will enable us to replicate the work packages in other African countries, where even less is known about PD, through our capacity building initiatives. We hope to facilitate further training in countries where there are few, or no, neurologists in collaboration with organisations such as the International Parkinson and Movement Disorders Society (MDS), as well as grow patient networks in these countries.

This grant is timely, as global recognition of PD and the public health challenge it poses builds. The work will contribute to broader initiatives, such as the WHO’s Intersectoral Global Action Plan on epilepsy and other neurological disorders [2] and align with the recommendations outlined in the Parkinson Disease technical brief [16].

### Supplementary Information

**Additional file 1.** 

**Additional file 2.** 

**Additional file 3.** 

**Additional file 4.** 

**Additional file 5.** 

**Additional file 6.**

## Data Availability

Not applicable.

## Abbreviations

ASAP: Aligning Science Across Parkinson’s
AUROC: Area Under the Receiver Operating Characteristic
CEI: Community Engagement and Involvement
CISI: Clinical Impression of Severity Index
ESP: Extra-cellular Synucleinopathic Protein
ESS: Epworth Sleepiness Scale
EQ5D 5L: EuroQol 5 Dimensions
GHRG: Global Health Research Group
GP2: Global Parkinson’s Genetics Program
GWAS: Genome-Wide Association Study
HADS: Hospital Anxiety and Depression Scale
IDEA: Intervention for Dementia in Elderly Africans
LMICs: Low- and Middle-Income Countries
LRRK2: Leucine Rich Repeat Kinase 2
MDS: International Parkinson and Movement Disorders Society
MERQ-PD: Mini-Environmental Risk factor Questionnaire-Parkinson’s disease
MJFF: Michael J Fox Foundation
MoCA: Montreal Cognitive Assessment
NIH: National Institutes for Health
NIHR: National Institute for Health and Care Research
NMP: NeuroMotor Pen
NMSS: Non-Motor Symptoms Scale
OCT: Optical Coherence Tomography
OTU: Operational Taxonomic Units
PD: Parkinson’s disease
PDQ8: Parkinson’s Disease Questionnaire
PDSS: PD Sleep Scale
PwP: People with Parkinson’s disease
QALY: Quality-Adjusted Life Year
QUIP-RS: Questionnaire for Impulsive-Compulsive disorders in PD Rating Scale
RBDSQ: Rapid eye movement sleep Behaviour Disorder Questionnaire
RCT: Randomised Controlled Trial
REDCap: Research Electronic Data Capture
SCOPA-AUT: Scales for Outcomes in PD Autonomic
SDGs: Sustainable Development Goals
SNP: Single Nucleotide Polymorphism
SSA: Sub-Saharan Africa
SPIRIT: Standard Protocol Items: Recommendations for Interventional Trials
SPSS: Statistical Package for Social Sciences
TraPCAf: Transforming Parkinson’s Care in Africa
UKPDBB: UK Parkinson’s Disease Brain Bank
UPDRS: Unified Parkinson’s Disease Rating Scale
WHO: World Health Organization
