# Supplementary material for: Transforming Parkinson's Care in Africa (TraPCAf): protocol for a multimethodology National Institute for Health and Care Research Global Health Research Group project
Source: BMC Neurol. 2023 Oct 19;23:373. doi: 10.1186/s12883-023-03414-0 (PMC10585779; doi:10.1186/s12883-023-03414-0)
Supplement: Supplementary file 1 — Additional file 1. [file 12883_2023_3414_MOESM1_ESM.pdf]

Healthcare resource use questionnaire*

***based on resource Utilisation in Dementia (RUD) Questionnaire**

Initial visit

1. During the last 30 days, how many times were you admitted to a hospital (for more than 24 hours)?

None ☐

Yes ☐ If yes, number of times: _______

2. If you were admitted to hospital during the last 30 days, please specify the total number of nights spent in each type of ward.

Geriatric ______

Psychiatric _______

Internal medicine _______

Surgery _______

Neurology _______

General ward _______

Other _______

3. During the last 30 days, how many times did you receive care in a hospital emergency room (for less than 24 hours)?

Number of times: ______

4. During the last 30 days, consider how many times did you visit a doctor, physiotherapist, psychologist or other health care professional. Please specify the number of visits for each type of care received. (*NB. Please do not include the neurologist visited as part of prevalence study)

☐ Did not visit any of these healthcare professionals during the last 30 days

General practitioner _______

Geriatrician ______

Neurologist* ______

Psychiatrist ______

Physiotherapist ______

Occupational therapist ______

Social worker ______

Psychologist ______

Nurse ______

Other ______ and please specify profession: ______________________

5. Have you accessed any care from traditional medicine/local medicine in the last 30 days?

Yes ☐ If yes, how much did it cost: ______

No ☐

6. Have you accessed any care from a faith healer in the last 30 days?

Yes ☐ If yes, how much did it cost: ______

No ☐

7. Provide details of all visits to traditional/local medicine/faith healer received in last 30 days.
