# Supplementary material for: Transforming Parkinson's Care in Africa (TraPCAf): protocol for a multimethodology National Institute for Health and Care Research Global Health Research Group project
Source: BMC Neurol. 2023 Oct 19;23:373. doi: 10.1186/s12883-023-03414-0 (PMC10585779; doi:10.1186/s12883-023-03414-0)
Supplement: Supplementary file 2 — Additional file 2. [file 12883_2023_3414_MOESM2_ESM.pdf]

Topic guide for semi-structured interviews with caregivers

Participant number: ______________

Date of interview: ________________

Location (country): _______________

Location (place of interview): __________________

Duration: _____________

Voice recorded: Yes No

Introduction

Thank you for agreeing to take part in this interview. I'm [researcher name] and I am working on the project 'Transforming Parkinson's Care in Africa'. We are conducting this qualitative study to understand people's experiences of living with, or caring for, someone with Parkinson's disease in [place]. Everything you say will be anonymous, even from the research team, so be as honest as you can. There are no right or wrong answers. We will also be asking you some basic questions about things like your age, and who you live with.

The interview should last about an hour but if you'd like to stop before then, that's fine. If I ask anything that you are not comfortable answering please let me know and we can move on to a different question or stop the interview. With your permission, I will be recording the interview. This will allow us to transcribe the interview so we can analyse it later.

[Ask for verbal consent to record]

Is there anything you'd like to ask me before we start the interview?

Demographic questions

About you

Q1. Age (number)

Q2. Gender (options)

Q3. Marital status (options)

Q4. No. children (number)

Q5. No. grandchildren (number)

Q6. How many people do you live with? (number)

Q7. Currently engaged in paid work? (yes/no)

Q8. What sector? (options)

Q9. Pension scheme? (yes/no)

Q10. Health insurance? (yes/no)

Q11. Any health conditions? (open question)

Interview

Firstly, could you tell me about your life before [person with Parkinson's] got Parkinson's?

> Probes: what kind of work; when did caring begin; what did people think/say

Could you tell me about what it's like now caring for someone with Parkinson's?

> Probes: affected work; how has it impacted life; relationships with friends; perceptions about disease; what kind of tasks/care

Could you tell me about your family?

> Probes: support; relationships with family; caregiver; role within family

Could you tell me about the services/treatment you've accessed for [person with Parkinson's]?

> Probes: diagnosis; neurologists; medication; traditional/faith healer; services available; experience of end of life if applicable

End

Thank you so much for giving up your time to take part in this research and for revealing some [emotional] information.

Is there anything else you'd like to talk about that you think is important that we haven't covered?

Thank you again, this recording will be kept safe and will be transcribed over the next few weeks and made anonymous.

Topic guide for semi-structured interviews with people with Parkinson's disease

Participant number: ______________

Date of interview: ________________

Location (country): _______________

Location (place of interview): __________________

Duration: _____________

Voice recorded: Yes No

Introduction

Thank you for agreeing to take part in this interview. I'm [researcher name] and I am working on the project 'Transforming Parkinson's Care in Africa'. We are conducting this qualitative study to understand people's experiences of living with Parkinson's disease in [place]. Everything you say will be anonymous, even from the research team, so be as honest as you can. There are no right or wrong answers. We will also be asking you some basic questions about things like your age, and who you live with.

The interview should last about an hour but if you'd like to stop before then, that's fine. If I ask anything that you are not comfortable answering please let me know and we can move on to a different question or stop the interview. With your permission, I will be recording the interview. This will allow us to transcribe the interview so we can analyse it later.

[Ask for verbal consent to record]

Is there anything you'd like to ask me before we start the interview?

Demographic questions

<u>About you</u>

Q1. Age (number)

Q2. Gender (options)

Q3. Marital status (options)

Q4. No. children (number)

Q5. No. grandchildren (number)

Q6. How many people do you live with? (number)

Q7. Currently engaged in paid work? (yes/no)

Q8. What sector? (options)

Q9. Pension scheme? (yes/no)

Q10. Health insurance? (yes/no)

Q11. Other diagnosed health conditions? (open question)

<u>About your Parkinson's</u>

Q12. When did you first notice symptoms of Parkinson's? (year)

Q13. When were you diagnosed with Parkinson's? (year)

Q14. Have you ever taken medication for Parkinson's? (yes/no)

Q15. When did you start treatment? (year)

Q16. What medications are you taking for Parkinson's? (list)

<u>About your care</u>

Q17. Do you have someone you identify as a main carer? (yes/no)

Q18. What relation is this person to you? (options)

Q19. How old is this person? (age)

Interview

Firstly, could you tell me about your life before you had Parkinson's?

> Probes: what kind of work; how noticed symptoms; why sought doctor; what did people think/say

Could you tell me about what it's like living with Parkinson's now?

> Probes: affected work; how has condition progressed; relationships with friends; perceptions about disease

Could you tell me about the treatment you've had for Parkinson's?

> Probes: neurologists; medication; traditional/faith healer; services available

Could you tell me about your family?

> Probes: relationships with family; caregiver; role within family;

End

Thank you so much for giving up your time to take part in this research and for revealing some [emotional] information.

Is there anything else you'd like to talk about that you think is important that we haven't covered?

Thank you again, this recording will be kept safe and will be transcribed over the next few weeks and made anonymous.

Topic guide for semi-structured interviews with healthcare professionals and policy makers

Participant number: ______________

Date of interview: ________________

Location (country): _______________

Location (place of interview): __________________

Duration: _____________

Voice recorded: Yes No

Introduction

Thank you for agreeing to take part in this interview. I'm [researcher name] and I am working on the project 'Transforming Parkinson's Care in Africa'. We are conducting this qualitative study to understand healthcare professionals and policy makers experience of Parkinson's disease in [place]. Everything you say will be anonymous, even from the research team, so be as honest as you can. There are no right or wrong answers. We will also be asking you some basic questions about the services you offer to people with Parkinson's.

The interview should last about an hour but if you'd like to stop before then, that's fine. If I ask anything that you are not comfortable answering please let me know and we can move on to a different question or stop the interview. With your permission, I will be recording the interview. This will allow us to transcribe the interview so we can analyse it later.

[Ask for verbal consent to record]

Is there anything you'd like to ask me before we start the interview?

Demographic questions

Q1. Profession (open question)

Q2. Private/public (if applicable)

Q3. How many people with Parkinson's do you see/treat per month? (open)

Q4. Rate knowledge about Parkinson's? (likert)

Interview

Firstly, could you tell me about your experience with Parkinson's disease?

Probes: knowledge/understanding about Parkinson's; experience in practise; any training

Could you tell me about your interactions with people with Parkinson's (if applicable)?

Probe depending on profession: follow up in clinic; prescribing medicine; dispensing medicine; alternative treatment; prayers; local hospital

Could you tell me about the services that people with Parkinson’s can access?

Probes: neurologists; medication; traditional/faith healer; services available; change over time; future need; palliative care or end of life care

End

Thank you so much for giving up your time to take part in this research and for revealing some [interesting] information.

Is there anything else you’d like to talk about that you think is important that we haven’t covered?

Thank you again, this recording will be kept safe and will be transcribed over the next few weeks and made anonymous.
