# Supplementary material for: Transforming Parkinson's Care in Africa (TraPCAf): protocol for a multimethodology National Institute for Health and Care Research Global Health Research Group project
Source: BMC Neurol. 2023 Oct 19;23:373. doi: 10.1186/s12883-023-03414-0 (PMC10585779; doi:10.1186/s12883-023-03414-0)
Supplement: Supplementary file 3 — Additional file 3. [file 12883_2023_3414_MOESM3_ESM.pdf]

Microbiome

SECTION A: Medication, herbs and supplements

			**Yes**	**No**
1.		Have you taken **antibiotics** *in the past 6 months*?	☐	☐
	a.	If yes, which ones (or what was it for)?	______________________	
	b.	If yes, when was the last time you took them?	______________________	
2.		Have you taken **anti-malarial** drugs *in the past 6 months*?	☐	☐
	a.	If yes, when was the last time you took them?	______________________	
3.		Have you taken **deworming** medication *in the past 6 months*?	☐	☐
	a.	If yes, when was the last time you took them?	______________________	
4.		Do you regularly take traditional herbs?	☐	☐
	a.	If yes, what herbs do you take?	______________________	
	b.	If yes, how often do you take the herbs?	☐ Daily ☐ Weekly ☐ Monthly<br>☐ Less often, specify ______________	

			**Yes**	**No**
5.		Do you regularly take supplements?	☐	☐
	a.	If yes, which supplements do you take?	______________________	
	b.	If yes, how often do you take them?	☐ Daily ☐ Weekly ☐ Monthly<br>☐ Less often, specify ______________	

SECTION B: Dietary habits

6. Please tick the box that best describes your consumption of each food type on average **in the past year**:

Food type	Never/less than once per month	1-3 times per month	1-2 times per week	3-5 times per week	Every day or almost every day
Dairy (e.g., milk, butter, cheese)	☐	☐	☐	☐	☐
Meat (e.g., chicken, beef)	☐	☐	☐	☐	☐
Fish (any type, e.g., red snapper, tilapia)	☐	☐	☐	☐	☐
Fruits (e.g., mango, banana)	☐	☐	☐	☐	☐
Vegetables (e.g., spinach, cassava)	☐	☐	☐	☐	☐
Cereal, bread, starches (e.g., maize, ugali, grains, potato)	☐	☐	☐	☐	☐
Oils (e.g., frying in vegetable fat)	☐	☐	☐	☐	☐
Carbonated soft drinks (e.g., soda)	☐	☐	☐	☐	☐
Added sugar (e.g., sugar in tea)	☐	☐	☐	☐	☐
Alcohol (e.g., beer, spirits)	☐	☐	☐	☐	☐

SECTION C: Water sources

7. What is the *main source* of the water you consume regularly?

☐ Bottled ☐ Borehole ☐ Rain ☐ Well ☐ Mains

☐ River/stream ☐ Sachet ☐ Tap ☐ Other, please specify ___________________

8. Is the water source private or communal? ☐ Private ☐ Communal

☐ Not applicable
