# Supplementary material for: Transforming Parkinson's Care in Africa (TraPCAf): protocol for a multimethodology National Institute for Health and Care Research Global Health Research Group project
Source: BMC Neurol. 2023 Oct 19;23:373. doi: 10.1186/s12883-023-03414-0 (PMC10585779; doi:10.1186/s12883-023-03414-0)
Supplement: Supplementary file 4 — Additional file 4. [file 12883_2023_3414_MOESM4_ESM.pdf]

TraPCAf pesticides use and exposure

		Yes	No
1.	Do you grow your own fruit/vegetables to eat?	☐	☐
	**If answer <u>no</u> to q1, go to <u>q2</u>.**		
	**If answer <u>yes</u> to q1, answer following:**		
a.	Do you use pesticides on them?	☐	☐
b.	Do you wash them before eating?	☐	☐
c.	Do you cook vegetables before eating?	☐	☐
2.	Do you wash or cook other fruit/vegetables that you buy to eat?	☐	☐
3.	Do you own a cow/goat/sheep?	☐	☐
	**If answer <u>no</u> to q3, go to <u>q4</u>.**		
	**If answer <u>yes</u>, answer following:**		
a.	Do you drink its milk?	☐	☐
4.	Have you worked with pesticides in the past?	☐	☐
5.	Do you currently work with pesticides?	☐	☐
	**If answer <u>no</u> to q4 and 5, go to <u>q6</u>.**		
	**If answer <u>yes</u> to q4 or 5, answer following:**		
a.	How many years did you work/have you worked with pesticides for?	______	
b.	How old were you when you first started working with pesticides?	______	
c.	Where do you mostly buy pesticides from?	____________________	

		Yes	No
d.	Do pesticides come with instructions for use?	☐	☐
e.	What are the names of the pesticides you use?	________________________	
f.	When do you apply the pesticides? ☐ Before planting ☐ After planting ☐ Both		
g.	How many times do you apply pesticides to a crop?	______	
h.	Do you wear protective clothing when using pesticides?	☐	☐
i.	Do you wear a mask or goggles?	☐	☐
j.	Do you mix the pesticides with your hands?	☐	☐
k.	What do you do with the pesticide containers after the pesticides are finished?	________________________<br>________________________	
6.	Does your neighbour apply pesticides to their crops?	☐	☐
		☐ Don't know	
7.	Do you sleep with an insecticide treated mosquito net?	☐	☐
