# Supplementary material for: Transforming Parkinson's Care in Africa (TraPCAf): protocol for a multimethodology National Institute for Health and Care Research Global Health Research Group project
Source: BMC Neurol. 2023 Oct 19;23:373. doi: 10.1186/s12883-023-03414-0 (PMC10585779; doi:10.1186/s12883-023-03414-0)
Supplement: Supplementary file 5 — Additional file 5. [file 12883_2023_3414_MOESM5_ESM.pdf]

ACMD Video protocol for Neurological exam

General notes:
Consider background and appearance of contrast on video. If possible film against blue/white background unless patient with white/light clothes. Please ask patient to remove socks and shoes.

Please Include in the Video:

1. **Top of head to upper shoulders in frame:**
   •Voice sample reading 3 lines of rainbow passage in translation or other cultural appropriate similar item that draws range of pitch. Consider microphone by pt if hypophonic (20 seconds)

2. Zoom IN to **face in frame:**
   •Saccades: Look to left (5 seconds), look to right (5 seconds), look up (5 seconds), look down (5 seconds)
   •Smooth pursuit: Follow finger to left (5 seconds), to right (5 seconds), to up (5 seconds), to down (5 seconds)
   - Smile/show teeth (5 seconds)
   - Protrude tongue (10 seconds)

3. Zoom OUT from **top of head to waist in frame**
   • Primary sitting position at rest (10 seconds)
   - Extension of both hands with elbows extended (10 seconds)
   - Extension of both hands with elbows flexed, fingers extended, hands positioned near mouth (10 seconds)
   - Finger to nose right hand (10 seconds)
   - Finger to nose left hand (10 seconds)

   • Naming animals (20 seconds) (distraction to draw out resting tremor or other suppressed movement disorder)
   • Finger tapping R (10 seconds), finger tapping L (10 seconds)
   • Pronate/supinate R hand (10 seconds), pronate/supinate L hand (10 seconds)

4. Zoom OUT to keep **waist to feet in frame**
   a. Tap right toe (10 seconds) tap left toe (10 seconds)
   b. Stomp right heel (10 seconds) stop left heel (10 seconds)
   c. Heel to shin right (10 seconds)
   d. Heel to shin left (10 seconds)

5. Zoom OUT to top of **head to toes, patient seated**
   a. Examiner assesses rigidity right wrist, elbow shoulder
   b. Left UE
   c. Right LE ankle and knee
   d. Left LE

    e. Examiner rates each
        i. 0- none
        ii. 1- mild
        iii. 2- moderate
        iv. 3- severe
        v. 4-fixed/nearly fixed

6. **Position chair side ways and film from side, head to toe in frame**
    a. Stand with both arms crossed (10 seconds)

7. **Head to toe in frame, patient with back to camera**
    a. Arms down and causally walk 10 feet, turn and return 10 feet
        i. Repeat x 1

8. Filming from looking above to below face patient **head to upper torso in frame with patient sitting to write on hard surface**
    a. Archimedes spiral right hand
    b. Archimedes spiral left hand
    c. Straight line between 2 dots right hand
    d. Straight line between 2 dots left hand
