# Supplementary material for: Transforming Parkinson's Care in Africa (TraPCAf): protocol for a multimethodology National Institute for Health and Care Research Global Health Research Group project
Source: BMC Neurol. 2023 Oct 19;23:373. doi: 10.1186/s12883-023-03414-0 (PMC10585779; doi:10.1186/s12883-023-03414-0)
Supplement: Supplementary file 6 — Additional file 6. [file 12883_2023_3414_MOESM6_ESM.pdf]

Women and Parkinson's

1. When was your Parkinson's diagnosed?

☐ While still having periods ☐ During pregnancy ☐ Up to 1 year after pregnancy

☐ While going through perimenopause* ☐ After menopause ☐ Unknown

**transitional period before menopause that can last for years*

2. What life phase currently applies to you?

☐ Still menstruating ☐ Post-menopausal

☐ On birth control but do not experience periods ☐ Unknown

☐ Going through perimenopause/menopause

3. If menstruating:

		**Yes**	**No**	**Unknown**
a.	Has your Parkinson's doctor asked you about your menstrual cycle and whether it affects your Parkinson's symptoms?	☐	☐	☐
b.	Has your menstrual cycle changed since the onset of Parkinson's symptoms?	☐	☐	☐
c.	Do your Parkinson's symptoms change in relationship to your menstrual cycle?	☐	☐	☐
d.	Do your Parkinson's symptoms related to movement change during your period?	☐	☐	☐
	If **yes**, do they get better or worse?	______________________		
e.	Do your Parkinson's symptoms related to thinking and mood change during your period?	☐	☐	☐
	If **yes**, do they get better or worse?	______________________		

f. During which phase of your menstrual cycle do your Parkinson's symptoms change?

☐ The week before a period ☐ Just before a period

☐ At the start of a period ☐ At the end of a period

4. Pregnancies:

a. How many pregnancies have you had? _______________

b. How many live births have you had? _____________

c. Have you had any pregnancies after being diagnosed with Parkinson's? ☐ Yes ☐ No
